# Polycyclic aromatic hydrocarbons in sediments of Hooghly River Mouth and Sundarbans Wetland, West Bengal, India

**DOI:** 10.1007/s10661-025-14763-3

**Published:** 2025-11-05

**Authors:** Maria Toscanesi, Michele Arienzo, Luciano Ferrara, Priyanka Mondal, Muthuswamy Ponniah Jonathan, Santosh Kumar Sarkar, Carlo Donadio, Marco Trifuoggi

**Affiliations:** 1 Dipartimento Di Scienze Chimiche, Università Degli Studi Di Napoli Federico II, Complesso Universitario Di Monte Sant’Angelo, Via Cintia 26, 80126 Naples, Italy; 2 Dipartimento Di Scienze Della Terra, Dell’Ambiente E Delle Risorse, Università Degli Studi Di Napoli Federico II, Complesso Universitario Di Monte Sant’Angelo, Via Cintia 26, 80126 Naples, Italy; 3 Department of Marine Science, University of Calcutta, 35 Ballygunge Circular Road, Calcutta, 700019 India; 4 Centro Interdisciplinario de Investigaciones y Estudios Sobre Medio Ambiente y Desarrollo, Instituto Politécnico Nacional, Calle 30 de Junio de 1520, Barrio La Laguna Ticomán, C.P. 07340 Del. Gustavo A. Madero, Ciudad de Mexico Mexico

**Keywords:** Hooghly Estuary, Sundarbans Mangrove Wetland, Polycyclic aromatic hydrocarbons, Sediment pollution, PAHs source diagnostic indexes, Factorial analysis, Carcinogenicity-mutagenicity, Ecological Risk assessment

## Abstract

The study compared the pollution level of 16 priority polycyclic aromatic hydrocarbons (PAHs) in the superficial sediments of the low stretch of the Hooghly River, LSHR, with that of the Sundarbans wetland, SW. Mean total PAHs levels were concerning, with mean individual loads exceeding legal limits and literature references, indicating a worsening scenario compared to past surveys. The mean of ∑PAHs in LSHR was significantly higher than in the SW, 2,106 ng/g vs. 1179 ng/g, with a hot spot of 13,785 ng/g. Thus, it is especially in the river location that contaminants accumulate with five compounds concentrating at higher rates compared to the natural area, 103% (PHE), 86% (FLT), 122% (PYR), 156% (CHR), and 1500% (DhA). An evident dominance of the heavy pool (4 to 6 rings) was also observed with a sixfold higher load with respect to the light pool (2 to 3 rings), 1,413 vs. 266 ng/g. PAHs source screening by diagnostic ratios revealed the dominance of pyrogenic sources. Most of sediments of the low stretch present high toxicity risk, and urgent attenuation measurements are needed to improve the environmental state of the river and nearby wetland ecosystems.

## Introduction

Wetlands make up 6% of the planet's surface and offer a wide range of ecosystem services, including preserving biodiversity, reducing flooding, protecting coastal areas from hurricanes, improving water quality, replenishing groundwater aquifers, acting as filter barriers, storing and transforming materials, and producing goods and food (Cherry, 2011). Depending on inflows, outflows, internal cycle rates, and anaerobic conditions, wetlands can act as sources, sinks, or transformers of carbon, nitrogen, phosphorus, iron, manganese, and sulfur (Villa & Bernal, 2018).

According to some writers (Cherry, 2011), wetland has an even greater economic value than lakes, rivers, forests, grasslands, and coastal estuaries. Because of this, numerous nations have implemented laws and policies aimed at protecting and restoring natural areas throughout the past century, such as the US's Clean Water Act of 1972 and the National Wetlands Policy Forum in 1988 (Hines, 2013). In the Iranian city of Ramsar, an international convention known as the Ramsar Convention was signed in 1971 and enacted in 1975 (Cherry, 2011). The treaty aims to conserve wetlands by requiring member nations to create wetland reserves, formulate national wetland policies, and identify one or more wetlands as areas of international significance. Many wetlands worldwide are severely damaged, despite these protection regulations due to unchecked population increase, industrialization, and economic development (De Groot et al., 2010).

The Sundarbans Mangrove Wetland (SMW) in India is a UNESCO World Heritage site and is regarded as a significantly sensitive habitat to anthropogenic hazards (Mondal et al., 2018). The SMW is a significant tide-dominating delta that is a component of the Hooghly River Estuary, a Ganges River distributary, formed by the alluvial deposits of the Ganga and Brahmaputra Rivers. In this location, freshwater overcome tidal flows during the monsoon season (June to September) and tropical cyclones, which frequently arise in the Bay of Bengal. Tidal flows outpace fluvial fluxes for approximately eight months of the year (Rogers et al., 2013).

**Fig. 1:**
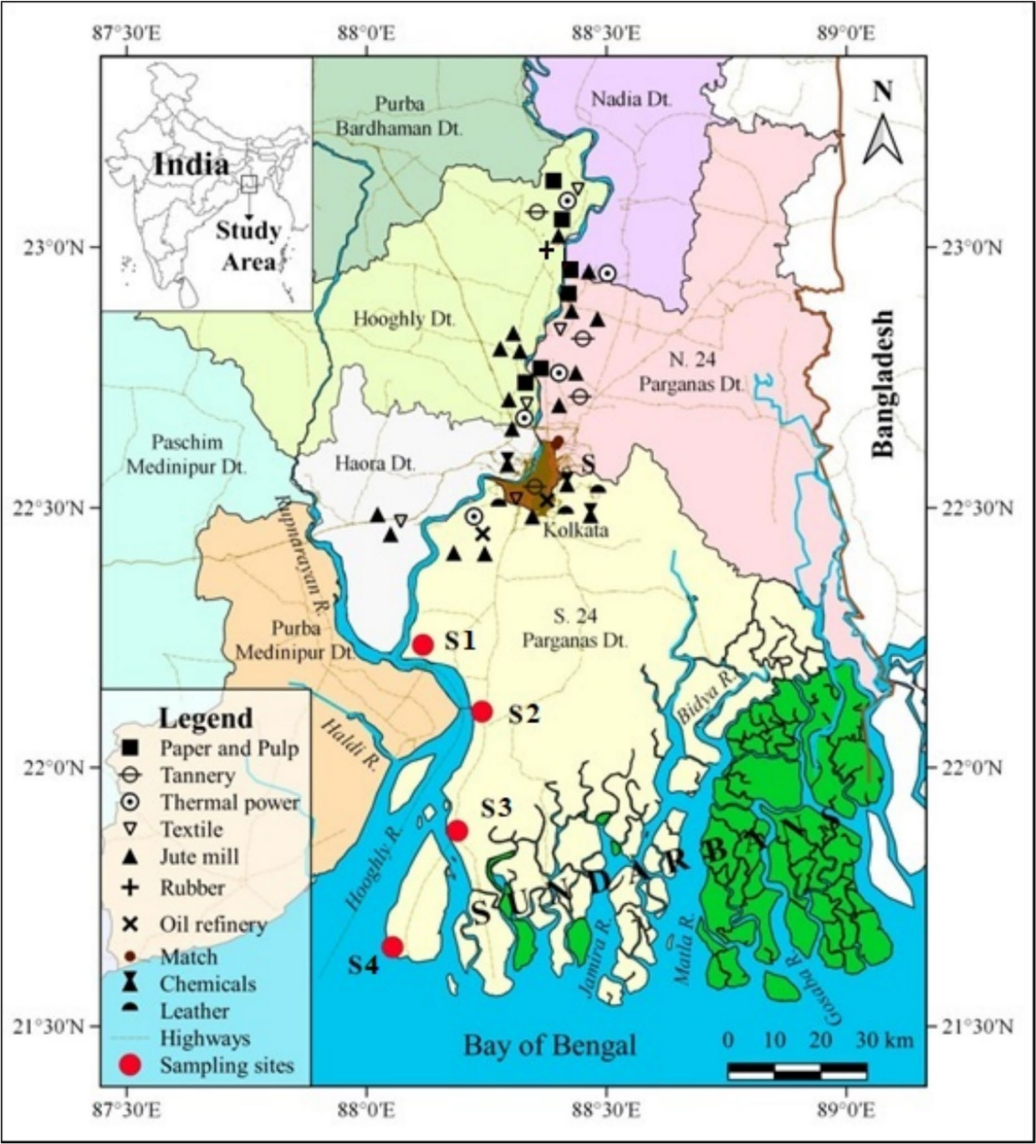
Map of sampling sites and of the anthropic pressure. The geographic coordinate system is WGS84

The Hooghly River, HR, has a catchment area of 6 10^4^ km^2^, and receives inputs from numerous cities built along its banks, as well as from chemical, paper, and pulp manufacturers, tanneries, thermal power plants, textile, and jute mills (Fig. 1). (Mondal et al., 2018; Sadhuram et al., 2005). Due to the wet Indian Summer Monsoon (June–September), the Hooghly River Estuary, HRE, is considered a "monsoonal estuary" (Shetye, 2011). It is distinguished by severe climatic gradients that propel intense biogeochemical processes, detritus erosion, and physico-chemical weathering (Garzanti et al., 2008, 2009).

According to Zuloaga et al. (2013), the Ganges River contributes 500 × 10^6^ tons of sediment annually to the delta, while the Brahmaputra contributes 700 × 10^6^ tons. The sediment load in the delta is somewhat constant. Hence, there are notable temporal variations in salinity, water velocity, and pollution loads due to the monsoon's high runoff and the dry season's eight-month low runoff.

Recent research, by Mitra et al. (2020), reports that the water quality of the HRE is poor, which is harmful to aquatic biota. The water's chemical properties vary based on the monsoonal seasons, flows, and the amount of silt carried into and deposited in the estuary. Outstanding levels of As, Cd, Ni, Pb, and U and Ni from anthropogenic sources, as well as a substantial ecological concern, were found in a recent study of the pollution state of HRE (Arienzo et al., 2023; Trifuoggi et al., 2022). Polycyclic aromatic hydrocarbons, PAHs, are one of the potentially hazards for SMW, being hydrophobic, very persistent and easily sorbing to suspended particulate matter and sediments (Zuloaga et al., 2013). In the marine environment PAHS accumulate from multiple sources: oil spills, ship traffic, urban runoff, garbage discharges, automobile and industrial stack emissions, and atmospheric fallout (Hassan et al., 2015). Of less importance is the natural sourcing of PAHs as from volcanic activities, wood fires, and moorland fires (Abdel-Shafy & Mansour, 2016).

Because of their harmful effects, PAHs are high-priority pollutants for the U.S. Environmental Protection Agency (USEPA) (Chen et al. 2012). According to the literature, high molecular weight (HMW) PAHs have teratogenic, mutagenic, and carcinogenic effects, while low molecular weight (LMW) PAHs have high and acute toxicity effects (Hassan et al., 2015). Very few studies on PAHs contamination of HR and SMW have been carried out (Balu et al., 2020; Dominguez et al., 2010; Zuloaga et al., 2013).

The study aimed to determine in the surface sediments of the last stretch of HR, LSHR, and SMW (1) the pollution and distribution of 16 priority polycyclic aromatic hydrocarbons and compare them with existing regulations and previous studies; (2) define potential sources of pollutants, either natural or anthropogenic, using statistical techniques such as diagnostic ratios;(3) assess the degree of ecological risk associated with PAHs in river and wetland sediments. The results will help in developing effective pollution control plans. Research on the temporal and spatial evolution of PAHs with seasonal events will continue on larger sections of the Hooghly River and SMW.

## Materials and methods

### The study area

The HR, which encompasses a basin of 6 10^4^ km^2^ and is heavily anthropized (Mondal et al., 2018), is a shallow-depth habitat with a mean depth of ~ 6 m (Mitra et al., 2017; Sadhuram et al., 2005). Its latitude is 87°55′01"N to 88º48′04"N and its longitude is 21º29′02"E to 22º09′00"E (Fig. 1). The existence of the SMW delta, formed by alluvial-marine deposits, is what defines HRE. Large tides, seasonal monsoons, and tropical cyclones, that pass across the Bay of Bengal, impact sediments offshore (Rogers et al., 2013).

### Sampling procedure

Surface sediments, 88 specimens, were taken in triplicate by a Van Veen grab at a nearly monthly frequency from November 2018 to May 2021, covering all monsoon seasons (Arienzo et al., 2024) at S1, Diamond Harbour, S2, Lot 8, belonging to LSHR and S3, Nurpur, and S4, Gangasagar, belonging to SMW, Fig. 1. The samples were placed in acid-rinsed polyethylene containers, kept at 4 °C, and then stored in the laboratory at −20 °C.

### Analytical procedure

pH, organic carbon C_org_ (%), CaCO_3_ (%), granulometry, micromorphology, and PAHs were determined on dry sediment samples, ϕ ≤ 2000 μm according to the procedure outlined by Ferrara et al. (2020), Arienzo et al. (2017), and Arienzo et al. (2024). The sixteen PAHs priority pollutants (US Epa, 1977) were determined: naphthalene, NAP, acenaphthylene, ACY, acenaphtene, ACE, fluorene, FLR, phenanthrene, PHE, anthracene, ANT, fluoranthene, FLT, pyrene, PYR, benzo(a)anthracene, BaA, chrysene, CHR, benzo(b)fluoranthene, BbF, benzo(k)fluoranthene, BkF, benzo(a)pyrene, BaP, dibenz(ah)anthracene, DhA, indeno[1,2,3-cd]pyrene, IPY, benzo(ghi)perylene, BgP. BaP is utilized as a toxicity standard due to its high carcinogenicity, and BaA, BaP, BbF, BkF, Chry, DBA, and IP are potential human carcinogens (IARC, 1987). The analysis of PAHs followed the IRSA CNR 25, slightly modified (Arienzo et al., 2017) by substituting acetone/n-hexane 1:1 v/v for cyclohexane and by employing an ultrasonic disruptor, Brason (US), with a power of 300 W in pulser mode, for a longer sonication time of three hours. The PAHs identification and quantitation were performed by a gas chromatograph (Shimadzu 2010 Plus, Japan) equipped by a fused silica HP5-MS capillary column (30 m × 0.25 mm i.d.) of 0.25 μm (Agilent Technologies, US) coupled with a mass spectrometer (MS-TQ8030-Shimadzu, Japan). Helium at 99.9% of purity was used as carrier gas and at a rate of 1.0 mL/min.

The program of the oven started at 80 °C for 2 min, then ramped up to 180 °C at 20 °C/min, and then reached 300 °C at 5 °C min for 9 min and the gas chromatograph was in spitless mode and the injector was kept at 280 °C. Perfluorotributylamine (PFTBA) was used to adjust the mass spectrometer in scan mode in accordance with the manufacturer's specifications. Quantitative determinations were made using the mass range m/z 50 and 300. The chromatograms were obtained, and data calculations were performed using GC Solution software. The composition and quantification of sample individual compounds were determined using an external calibration consisting of PAH standards.

For every site under investigation, samples were analysed three times. The recovery was calculated by analysing a certified sediment matrix containing the PAHs of interest (CRM104 Supelco). According to current methodologies, the recovery rate ranged from 57 to 130%. The approach demonstrated strong selectivity and specificity by achieving a good PAHs cleanup separation procedure with minimal coextract interference that could affect the accuracy of the result. For every PAHs under investigation, the range method of prediction to 95% of linear regressions was used to determine the detection limit (LOD) and limit of quantification (LOQ).

The average LOD and LOQ values that were determined were 3 and 10 ng/g, respectively. The limit of detection (LOD) was estimated using the range method based on 95% prediction intervals from the linear regression of the calibration curve. The limit of quantification (LOQ) was initially estimated as approximately three times the LOD, in line with common practice. The LOQ was further verified by subsequently analysing samples at this concentration to ensure acceptable levels of accuracy and precision. The quality assessment of the data was performed by interlaboratory ring tests. Unmixing PAHs in sediment samples to its sources classes was performed by diagnostic ratios and matrix factorization.

The toxicity of the PAHs mixture was calculated by determining the equivalent toxicity, TEQ, based on the BaP equivalents for the whole group of PAHs. BaP is classified as a group 1 carcinogen, whose metabolites are mutagenic and impede DNA replication (Mumtaz & George, 1995; Obini et al., 2013). TEQ was calculated by the toxicity equivalence factors, TEF, of the individual PAHs relative to BaP (US Epa, 1993, Nisbet & LaGoy, 1992). Similarly, the equivalent mutagenicity, MEQ, and the equivalent carcinogenicity, CEQ, were determined based on the mutagenic and carcinogenic equivalence factors relative to BaP and 2,3,7,8-tetrachlorodibenzodioxin (Ferrara et al., 2020). Furthermore, the ratio of the carcinogenic to total PAHs concentration, PAHscarc/ΣPAHs, was calculated (US Epa, 2008). The formula used to compute the total BaP toxicity Equivalent (TEQ) for all PAHs was TEQcarc = R (Ci x TEFcarc), where Ci was the concentration of each PAH (ng/g d.w.) and TEFi was its matching TEF.

The ecological risk of PAHs was determined by the calculation of the toxic equivalency factors based on the negligible concentrations, NCs, and maximum permissible concentrations, MPCs (Cao et al., 2010; Wu et al., 2011).

The following risk quotients were calculated:

1 $$R{Q}_{NCs}= {C}_{PAHs}/{C}_{QV(NCs)}$$

2 $$R{Q}_{MPCs}= {C}_{PAHs}/{C}_{QV(MPCs)}$$

where C_QV(NCs)_ and C_QV(MPCs)_ indicate the quality values of the NCs and MPCs of PAHs in the medium, respectively (Ferrara et al., 2020).

Principal component analysis was performed by STATISTICA v.5 (StaSoft Inc., Tulsa, OK, USA) as a confirmation model of PAHs fingerprints.

## Results and discussion

### Physico-chemical features of sediments

The locations of the studied areas are shown in Table 1, together with the geochemical characteristics of the sediments, pH, C_org_ (%), CaCO_3_%, and the textural percentage composition of sand, silt, and clay (%). The shape of the quartz silicate granules in the 384–177 µm range, the mean size (Mz), mode (Mo), standard deviation (σ_I_, sorting), skewness (Sk_I_, asymmetry coefficient), and kurtosis (K_G_, appointment coefficient), are displayed in Table 2.

**Table 1:** Sampling location and mean values of pH, C_org_, CaCO_3_, and grain size of sediments from LSHR and SMW. The range of variations is given in parentheses

Location	Sampling sites	Latitude N	Longitude E	pH	C_org_ (%)	CaCO_3_ (%)	Sand (%)	Silt (%)	Clay (%)	Class*
LSHR	S1-Nurpur	22°12′40”	88°04′16”	7.59 ± 0.33 (6.80–8.11)	0.39 ± 0.13 (0.15–0.65)	11.10 ± 1.59 (7.2–13.9)	7.45 ± 2.89 (2.1–13.4)	32.90 ± 9.03 (24.3–61.7)	59.60 ± 7.88 (36.1–68.5)	Silty clay
				(6.80–8.11)	(0.15–0.62)	(7.20–13.9)	(2.15–13.40)	(24.30 ± 61.70)	(36.15–68.50)	
	S2-Diamond Harbour	22°11′13”	88°11′24”	7.55 ± 0.24 (7.16–7.94)	0.47 ± 0.19 (0.12–0.84)	11.20 ± 2.12 (6.3–14.7)	8.73 ± 2.64 (4.4–13.7)	32.80 ± 10.40 (25.0–63.3)	58.40 ± 9.34 (31.7–66.0)	Silty clay
				(7.16–7.94)	(0.12–0.84)	(6.34.14.70	(4.36–13.70)	(25.00–63.30)	(31.70–66.01)	
SMW	S3-Lot 8	22°52′29”	88°10′09”	7.46 ± 0.23 (7.03–7.83)	0.42 ± 0.15 (018–0.76)	11.70 ± 3.05 (2.0–15.5)	10.40 ± 4.88 (0.9–20.1)	32.20 ± 9.44 (7.2–48.8)	58.30 ± 11.22 (31.1–87.5)	Silty clay
				(7.00–7.80)	(0.18–0.76)	(1.98–15.50)	(0.90–20.09)	(7.25–48.80)	(31.10–87.60)	
	S4-Gangasagar	22°38′24”	88°04′46”	7.44 ± 0.19 (7.13–7.91)	0.43 ± 0.20 (0.15–0.82)	9.98 ± 2.05 (6.37–15.5)	34.60 ± 11.60 (17.3–65.4)	28.40 ± 5.85 (17.0–40.4)	37.00 ± 9.55 (17.0–51.6)	sandy loamy clay
				(7.13–7.91)	(0.15–0.82)	(6.37–15.50)	(17.3–65.40)	(17.00–40.40)	(17.00–51.60)	

* Classification of sediment grain size (Folk & Ward, 1957)

**Table 2:** Granulometry and main statistical indices of sediments from LSHR and SMW

Location	Sampling sites	Mo	Mz	σ_I_ (ϕ^1^)	Sk_I_	K_G_
LSHR	S1 Nurpur	unimodal	4.482	0.610	0.445	1.186
	S2Diamond Harbour	unimodal	4.859	0.776	0.538	1.517
SMW	S3 Lot 8	unimodal	4.852	0.899	0.431	1.075
	S4 Gangasagar	unimodal	4.850	0.805	0.409	1.137

^1^ϕ = -log_2_ *D*_mm_, where *D* is the diameter of the grain in millimeters

Regarding pH, the reaction is generally close to the neutral or subalkaline range (7.44–7.59), with the mean pH at LSHR of 7.57 and at SMW of 7.45. There is a small geographical difference between the two areas. The highest pH value reaches a peak of 8.11 at S1 Nurpur, while S4 Gangasagar at SMW reported a top pH of 7.91. This trend reveals a moderate influence of marine waters that normally have a pH range of 8.08–8.33 (Marion et al., 2009) whereas most natural freshwaters have pH values of 6.5–8.0 (Radke, 2002). The organic carbon (C_org_%) load of the sediments seemed to be modest, with a mean range of 0.39–0.47%. S1 Nurpur sediments had the lowest C_org_%, richness (mean value 0.39%), and narrowest variation range (0.15–0.65) of all the sediments under study. These values seem to be lower than those reported by Balu et al. (2020) for Kakdwip, 6.7–8.3 C_org_%, which falls in the SMW area, and those detected in sediments from other coastal areas of India, such as the Gulf of Mannar (Jonathan et al., 2003), Cochin (Sunil Kumar, 1996), and Muthupet mangroves (Janaki-Raman et al., 2007). C_org_% appears consistent with data published by Subba Rao (1960), who observed extremely low levels of organic carbon in shelf sediments composed of clay and very fine silt found in the Krishna and Godavari basins on India's east coast. This discrepancy can be attributed to the fact that our data is averaged over a long period, spanning three years, and the progression of the three monsoon seasons. According to Trifuoggi et al. (2022), the monsoon season has caused the organic load of sediment to decrease because the river flooded and the finest sediments containing organic components were removed. Beside this, other possible causes regard differences in sampling season, sampling depth, or other site-specific environmental conditions.

The sediment organic load then recovered during the post-monsoon season through re-sedimentation and the deposition of organic material and subsequently fell once more throughout the pre-monsoon season. Furthermore, harsh environmental circumstances that impact tides and, consequently, the capacity of microbes to degrade sediment are likely to be the primary cause of the organic load in these places (Antizar-Ladislao et al., 2015; Chatterjee et al., 2007; Sarkar et al., 2004). Overall, there was a high mean content of CaCO_3_ (11.0%), with a clear decreasing pattern from LSHR to SMW (11.2–9.98%). The anomaly to detect higher loads of CaCO_3_ in S1 and S2 and S3 respect to S4 was likely caused by several factors, including river flowing through limestone areas, the transport and mixing of terrigenous fractions from continental sediments, and the prevalence of flood-related deposits in tropical environments (Carthew et al., 2003).

**Fig. 2:**
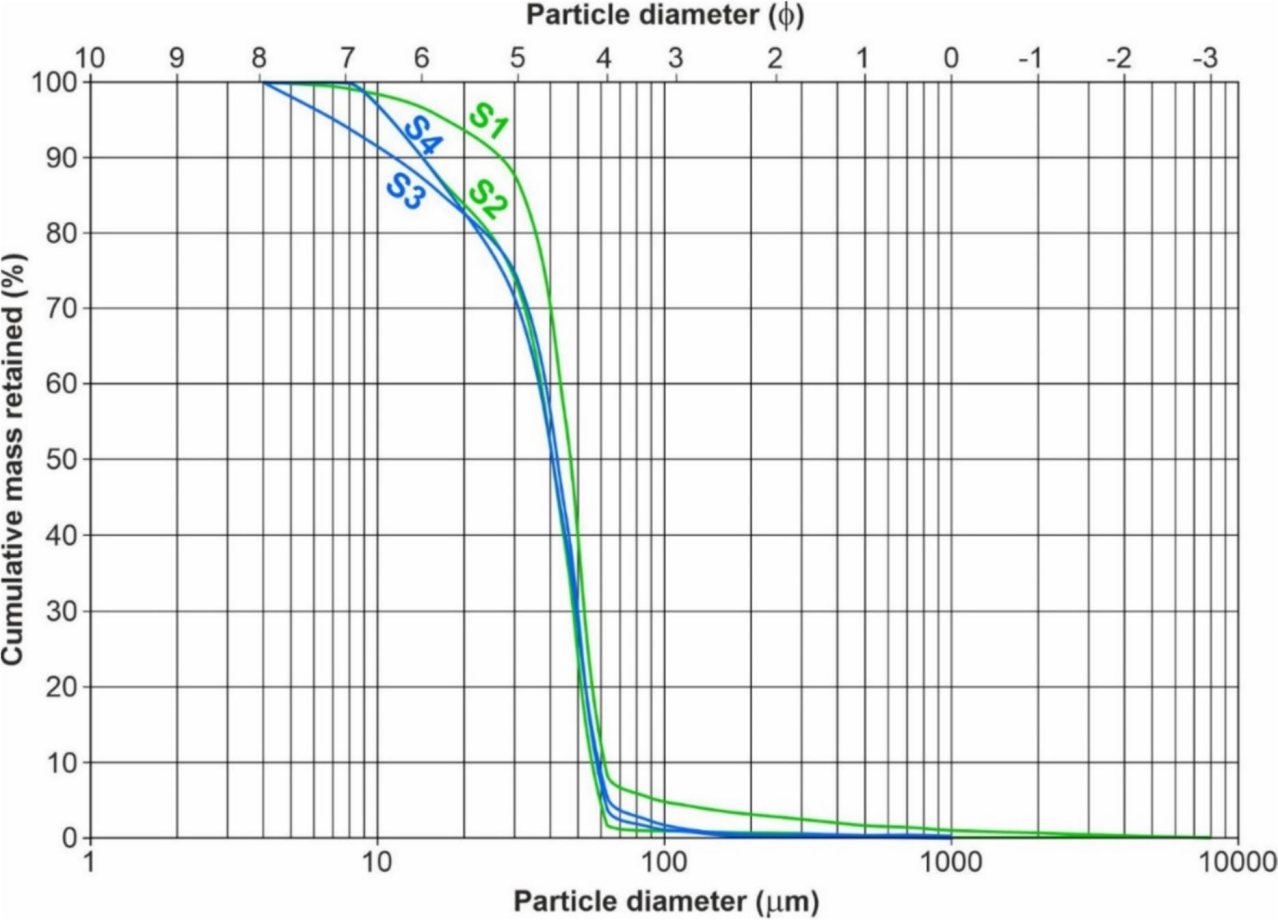
Diagram of the semilogarithmic granulometric curves of the four samples collected along the LSHR (S1 and S2, green) and SMW (S3 and S4, blue) fluvial-marine sectors. Units of grain diameter are both in microns and ϕ

Sediment texture properties, Table 1 and Fig. 2, reveal the predominance of the silty clay class except for the marine site of S4 being the class sandy silty clay according to the classification of Folk and Ward (1957). Considering the percentages of the single granulometric classes, a higher richness of clay at LSHR is evident compared to SMW, from a mean value of 59.02% to 47.64%. The lower clay content at S4 of 36.96% might be due to the confluence of HR with the Bay of Bengal, where currents move and mix fine particles with a recharge of coarser sediments from marine ingression with a mean sand load of 34.61% which is fourfold the mean load of the other sites of 8.87%. Unlike clay and sand, silt contents are constant with a narrow range of variation between 28.43% and 32.93% and higher loads in the LSHR up to 61.7% at S2.

The poor hydrodynamic regime is confirmed by the prevalence of finer silt and clay deposits in LSHR. Also, freshwater inflow is indicated by the finer particles that settle to the bottom when winds and currents decrease. The behaviour of S4, whose granulometric composition differs significantly from that of the other sites with higher loads of coarse components, should also be emphasized. This is consistent with the site's coastal marine location, where the movement of the water disturbs the sedimentation of fine particles.

The Mz of the surface sediments was lowest at S1 with a mean value of 4.482 φ (44.76 µm) and highest at S2, 4.859 φ (34.46 µm). If we average the data for the LSHR, a mean of 4.67 φ (39.28 µm) results, which is below the mean of the SMW, 4.851 φ (34.65 µm), and the coarsest sediments (> 3.5 φ or 90 µm) occur at S4 at the mouth of the estuary. The mean sorting coefficients, σ_I_, of the surface sediments at LSHR were 0.693 φ (1.61 µm) and 0.802 φ at SMW, indicating moderately well-sorted in both locations, likely due to a better distribution of the finer sediments. The grain size distribution, Sk_I_, was very fine skewed at both locations, with skewness varying from 0.538 φ (−0.538 µm) at S2 to 0.409 φ (−0.409 µm) at S4.

According to Liang et al. (2020), hydrodynamic conditions, topography features, sediment sources and characteristics have the greatest effects on the geographic distribution of grain-size parameters. The sediment samples are generally leptokurtic, with those having a value of 1.11–1.50 φ being leptokurtic, and those with values of 1.51–3.00 φ being very leptokurtic.

### Distribution of PAHs in sediments

Table 3 shows the concentrations, in ng/g, of PAHs in the four sampled sites of LSHR and SMW. The mean ∑PAHs in LSHR were significantly higher, ~ 78%, p < 0.05, than in SMW, 2106 ng/g vs. 1179 ng/g, with a higher peak in SMW regarding LSHR, 18,625 vs. 13,785 ng/g. It is especially in S2 that contaminants accumulate with an average value, of 3061 ng/g, which is about three times higher than in the other sites, ~ 1170 ng/g. The Hooghly River's elevated PAH content may be related to heavy boating, sewage and garbage discharges, and urban runoffs (Balu et al., 2020; Sarkar, 2016). The two regions under investigation exhibit distinct hydrodynamic regimes, wherein the SMW is impacted by the tidal input. Tidal asymmetry and mixing of flood and ebb flow result in sediment resuspension and more variability of PAH loads. Five individual compounds are at levels higher in LSHR than in SMW: PHE, 103%, FLT, 86%, PYR, 122%, CHR, 156% and DhA, 1512%. Overall, if we look at the distribution of hydrocarbons into light, LPAHs (2 to 3 rings), and heavy, HPAHs (from 4 to 6 rings) we note the clear dominance of the heavy pool, with an average of 1413 ng/g, which is about six times higher than that of light ones, 266 ng/g. This trend agreed with what was reported by Balu et al. (2020) who reported a mean total HPAHs level of 1.04 × 10^4^ ng/g and mean LPAHs levels of 2150 ng/g. Moreover, the HPAHs pool predominates in S2, 2902 ng/g, and S4, 1229 ng/g, whereas the mean LPAHs level remains constant, ~ 268 ng/g, over the four investigated sites. Data agree with those of Dominguez et al. (2010), Sarkar et al. (2012), Zuloaga et al. (2013), and Balu et al (2020) who reported a higher abundance of 4–6 ringed PAHs. In fact, in our survey FLT and PYR predominate, and this happens especially in LSHR.

**Table 3:** Mean, minimum and maximum concentration (ng/g), ERL, ERM, and AET of PAHs in sediments

PAHs	S1 mean	range	S2 mean	range	S3 mean	range	S4 mean	range	ERL	ERM	AET	^e^Italian limit
NAP	2.60	nd-37.6	0.528	nd −11.1	nd	nd	3.45	nd-75.9	160	2100	500	35
ACY	nd	nd	46.5	nd-276	1.16	nd-25.6	36.1	nd-529	16	500		
ACE	nd	nd	nd	nd	nd	nd	nd	nd	44	640	150	
FLR	2.45	nd-32.8	3.12	nd-29.2	nd	nd	0.450	nd-9.89	19	540	350	
PHE	258	nd-2315	146	nd-855	154	nd-2147	105	nd-1191	240	1500	260	
ANT	59.9	nd-286	109	nd-578	73.9	nd-315	63.0	nd-391	85	1100	300	45
^b^LPAHs	323		305		229		208		564	6380	1560	
FLT	417	nd-5006	739	nd-3369	363	nd-4321	256	nd-2515	600	5100	1000	110
PYR	214	nd-2629	580	nd-2440	185	nd-2147	170	nd-1794	665	2600	1000	
BaA	16.9	nd-269	371	nd-1642	22.1	nd-268	79.9	nd-1199	261	1600	550	
CHR	23.4	nd-270	302	nd-1266	31.2	nd-284	93.9	nd-1336	384	2800	900	
BbF	11.9	nd-80.7	249	nd-1456	19.5	nd-135	152	nd-2268				40
BkF	7.04	nd-59.1	161	nd-1028	10.1	nd-92.2	86.2	nd-1408				
BaP	16.8	nd-90.6	114	nd-1026	18.6	nd-82.1	105	nd-1633	430	1600	700	30
DhA	100	nd-2180	38.5	nd-302	1.53	nd-33.6	7.15	nd-136				
IPY	11.1	nd-52.8	171	nd-1299	18.2	nd-103	145	nd-2226				70
BgP	10.2	nd-52.8	176	nd-1084	23.8	nd-99.0	133	nd-2058				55
^c^HPAHs	829		2902		693		1229		2340	1.37 × 10^4^	4150	
ΣPAHs	1151	40–11093	3207	nd-13785	921	nd-9965	1437	nd-18625	2904	2.01 × 10^4^	5710	800
^d^PDPAH	108	nd-525	773	nd-4770	146	nd-702	544	nd-7834	675	4800	1500	

^a^ERL: effects range low value, ERM (Long et al., 1995): effects range medium value (Long et al., 1995); AET: apparent effects threshold (Kim et al., 1999); ^b^LPAHs: NAP, ACY, ACE, FLR, PHE, ANT; ^c^HPAHs: FLT, PYR, BaA, CHR, BbF, BkF, BaP, DhA, IPY, BgP; ^d^PDPAH: NAP, ANT, BbF, BkF, BaP, and BgP; ^e^Italian rules, law nr. 219/2010 (Ferrara et al., 2020)

Literature reports limited and unclear trend levels of PAHs along HRE and SMW. If the overall mean range of sedimentary PAHs was 921–3,061 ng/g the relative range of variation was quite wider, nd-18,625 ng/g. PAHs loads were mostly higher than those cited in Hoogly River sediments surveys: 48.6–1098 ng/g, reported by Binelli et al. (2008), 208–12,993 ng/g, Zuloaga et al. (2013), 132–2938 ng/g, observed by Dominguez et al. (2010) in the SMW, and fairly similar to the range 4880–20,000 ng/g outlined by Balu et al. (2020) for SMW. Thus, our data seem to suggest an escalation of the contamination by PAHs in the SMW over time. Compared with other Indian locations our PAHs load appears lower than 13–31,400 ng/g cited by Goswami et al. (2016) for the Adyar estuary, 5050–33,100 reported by Kumar et al. (2016) for Chitrapuzha River, and 17–134,134 ng/g Dhananjayan et al. (2012) for Mumbai Harbour. Our PAHs data range also appears significantly larger than those documented in the literature for estuaries located in other countries: the west coast of Tunisia, 40–655 ng/g (Ameur et al., 2010); the Brisbane River in Australia, 148–3079 ng/g (Duodu et al., 2017); the Chongqing River in China, 221–3205 ng/g (Lei et al., 2019);

Table 3 also displays the numerical sediment quality guidelines (SQGs), ERL, Effect Range-Low, ERM, Effective Range-Median, as proposed by Long et al. (1995) and AET, Apparent Effects Threshold, derived from the approach of Kim et al. (1999) and sediment quality standards for the Mediterranean coast (Ferrara et al., 2020). There is only one location, S2, where the mean PAHs load, 3,207 ng/g, surpassed the ERL limit of 2,904 ng/g. Here, mean ACY, PHE, and ANT levels surpassed up to three-fold the relative ERL threshold, as in the case of ACY, 46 vs. 16 ng/g. FLT and BaA exceeded the relative ERL by 23–42%. In the case of SMW, only maximum loads of contaminants, 18,625 ng/g, were ninefold the ERL and threefold the AET. The results of this study showed that the mean values of ∑PAHs in sediments were considerably lower than the ERM and ERL guidelines. Only at some punctual sites contaminants exceed the relative ERL and might create detrimental biological consequences (Kim et al., 1999; McDonald et al., 2000).

Among the individual PAHs, the levels of ANT, BbF, BkF, BaP, IPY, and BgP surpassed the relative legal limits set for Mediterranean coastal sediments, at S2 and S4, and with increments of two to four times the limits. Only BbF and BkF showed a much greater exceedance of the limits especially at S2 and up to eight times, 249 vs. 40 ng/g and 161 vs. 20 ng/g. ∑PAHs level appeared outstandingly high at all locations, with more worrying loads at S2 and S4 where ∑PAHs surpassed the legal limit of 800 ng/g of two to four times. Compared with the class of high sediment pollution level of the literature, Zakaria et al. (2002), 1001–5000 ng/g our data, 921–3207 ng/g, reveal how the studied sediments deserve urgent action for pollution containment.

However, the use of SQGs for individual compounds has limitations because in real field conditions, sediments are characterized by the simultaneous presence of complex mixtures of hydrocarbons, so their cumulative effect cannot be excluded (Binelli et al., 2008). Therefore, we also refer to ∑PAHs thresholds proposed by Swartz (1999) that divided the pool of total PAHs into three clusters: TEC, threshold effect concentration (2,900 ng/g), PEC, probable effect concentration (18,000 ng/g) and EEC extreme effect concentration (10,000 ng/g), calculated as arithmetic mean of values in each cluster. In our study, there was no evidence of mean total PAHs loads surpassing the above limits. A high positive correlation was observed between Corg_%_ and PAHs (r^2^ = 0.758). Similarly, other authors (Liu et al., 2017) report a favourable linear correlation (Liu et al., 2017) and relate this trend to the organic carbon sediment richness and their consequent more intricate pore structure and hydrophobicity, facilitating easier PAHs absorption. However, Sarkar et al., (2012, 2016) reported that for the SMW contrasting outputs, a weak and strong correlation exists between Corg_%_ and total PAHs. Sediment Corg_%_ is therefore not the sole factor affecting sediment PAH levels and distribution, but other factors should be considered. These factors regard the dynamic of currents, composition and deposition of sediments, input of PAHs, processes of resuspension and redeposition with meteorological or biological events, as well as microbial degradation of PAHs (Sarkar, 2016).

**Fig. 3:**
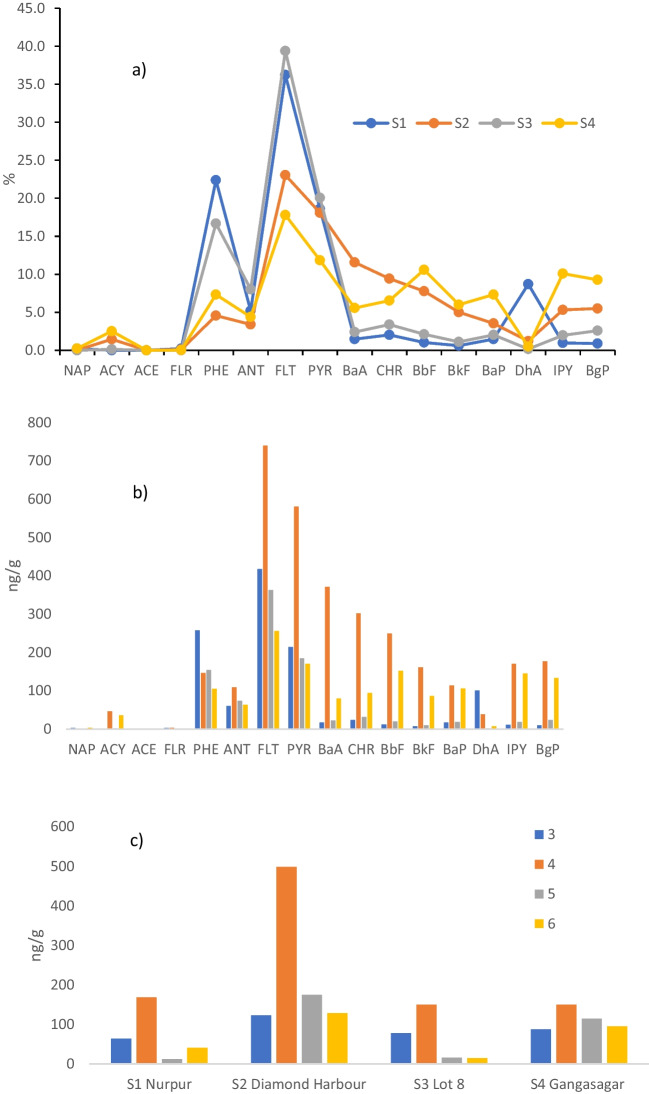
**a**) Percentage concentrations; **b**) spatial distributions and **c**) 3, 4, 5, 6 rings of PAHs (ng/g dry weight)

The relative percentage composition, the spatial distributions of PAHs and of the three, four, five, and six-ring compounds (ng/g dry weight) were reported in Fig. 3. Figure 3a evidenced a heterogeneous PAHs percentage composition in the studied area; similar patterns were displayed by S1 and S3, with PHE plus FLT making ~ 60% of the total pool, and by S2 and S4, with PHE plus FLT making ~ 25% of the total. The distribution profile of PAHs compounds drawn in Fig. 3b was also heterogeneous and quite identical only for ANT, FLT, and PYR, and scaled as follows: S2 > S1 > S3 > S4. This indicates that even SMW, quite far away from pollution sources, is impacted by the identical sources of pollution affecting S1 and S2 due to similar sources of contamination, transport, and deposition processes. The composition pattern of ring size, Fig. 3c, reveals that 4 aromatic rings (FLT, PYR, BaA, CHR) dominate at all sites and make up 41.7–65.1% of the mixture, followed by those with 5 rings (BbF, BkF, BaP, DhA) prevailing at S2 and S4 with a range of 18.3–24.4% of the total. The 2-ring (NAP) load was very low and varied between 0–0.24% whereas the 3 rings (ACY, ACE, FLU, PHE, ANT) displayed similar rates of 27.8–24.9% at S1-S3 and of 10.0–14.2% at S2-S4, revealing once again a similar source of pollution and deposition.

### PAHs sources (pyrogenic/petrogenic)

**Fig. 4:**
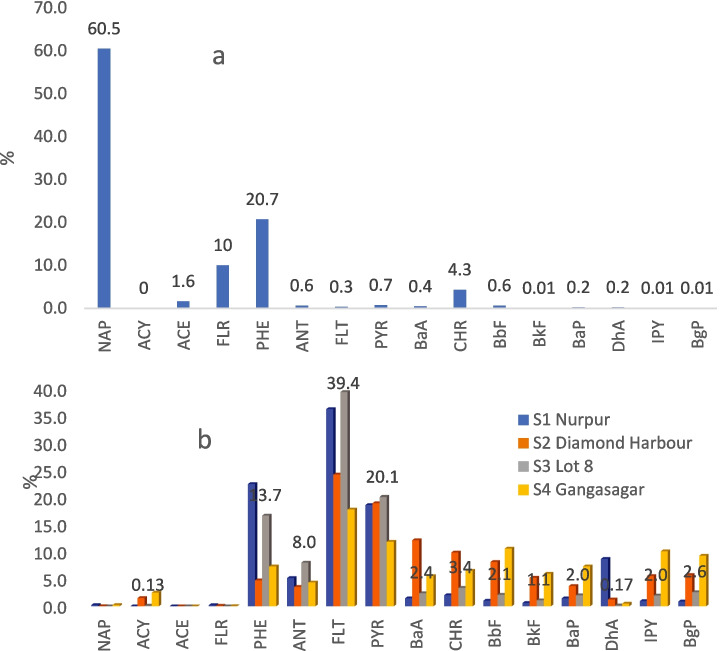
Relative percentage distribution of PAHs in a) crude oil (Kerr et al., 1999) and b) sediments of LSHR and SMW (mean of all the sampling sites)

A mixture's hydrocarbon distribution offers important insight into its history. PAHs from petroleum-derived (petrogenic) sources are more concentrated in components with lower molecular weights (two to three-ringed PAHs) while combustion-producing mixtures generated are primarily four to six-ringed PAHs, which are more thermodynamically stable and toxic and are indicators of pyrogenic source of contamination (Adeniji et al., 2018). Most molecular indexes, Table 4, indicated a pyrogenic origin with no significant differences between LSHR and SMW. ANT/(ANT + PHE) is one of the most useful and commonly used diagnostic reports to differentiate oil from a combustion source (Yunker et al., 2002). This index in all locations was mainly > 0.1, indicating the preponderance of the source of pyrogenic matter. This was also confirmed by the scant matching of the PAHs composition of the studied sediments with that of crude oil, with a clear dominance of PHE and FLT up to 40% (S1 and S3), Fig. 4. However, a net pyrogenic origin of the PAHs mixture from incomplete combustion-related sources was not observed since FLR/(FLR + PYR) and ∑COMB/∑PAHs also reveal petrogenic origin from hydrocarbon spills, being the indexes below the petrogenic threshold of 0.5 and 1, respectively.

**Table 4:** Diagnostic ratios determined at each sampling station

Molecular index	Location				Source	
	S1	S2	S3	S4	Petrogenic	Pyrogenic
PHE/ANT	4.31	1.34	2.08	1.67	> 15	< 10
CHR/BaA	1.39	0.81	1.41	1.17	< 0.4	> 0.9
BaP/BgP	1.65	0.64	0.78	0.79		> 0.6
FLT/PYR	1.95	1.27	1.96	1.50	< 1	> 1
ANT/(ANT + PHE)	0.188	0.427	0.325	0.374	< 0.1	> 0.1
FLT/(FLT + PYR)	0.661	0.560	0.663	0.600	< 0.4	≥ 0.5
FLR/(FLR + PYR)	0.011	0.005		0.003	< 0.5	> 0.5
IPY/(IPY + BgP)	0.521	0.492	0.433	0.521	< 0.2	> 0.5
BaA/(BaA + CHR)	0.419	0.551	0.415	0.460	< 0.2	> 0.35
^a^Ring456/∑PAHs	0.720	0.948	0.752	0.855	< 0.4	> 0.5
LPAHs/HPAHs	0.389	0.105	0.330	0.169	> 1	< 1
^b^∑COMB/∑PAHs	0.623	0.880	0.730	0.749	< 1	~ 1

^a^Ring456: Total 4–6 ring PAHs (mainly originated from combustion); ^b^∑COMB (FLT, PYR, BaA, CHR, BbF, BkF, BaP, BgP)

Table 5 provides the loading factors, total and cumulative variance generated by the principal component analysis, PCA, of pH, C_org_, CaCO_3_, sand, silt, and clay, and PAHs, of LSHR and SMW. The output shows for both areas, that one factor accounts for a great portion of the total variance, 41.9 and 57.05% with most of the heavy individual hydrocarbons possessing positive significant loads, p < 0.05, in the range of 0.73–0.93 for LSHR and 0.70–0.99 for SMW. Among low molecular weight compounds, only ACY for LSHR and NAP and ACY for SMW showed significant positive loads of 0.88 and 0.91, and 0.94, respectively. The increase of the number of considered factors up to 3 increased the total cumulative variance, > 70%, but with no grouping of positive significant loads. These data therefore seem to confirm the output generated by molecular ratios, highlighting the dominant influence of the HPAHs and hence of the pyrogenic source, whereas the role of LPAHs appears secondary and likely linked to the effect of heavy rain events, when flooding, addition, erosion, remix and transport of sediments also occur. In recent times, the Hooghly River has been seriously devastated due to the enormous pollution load from point and non-point sources (Roy et al., 2022). Various industries like cement, paint, tannery, dairy, paper and petrochemical and thermal power plant were built on the Hooghly River basin. It is likely that PAHs are sourced from domestic use of coal, charcoal, wood, rice husking, and coal power stations, as well as urban runoff, automobile emissions, and river discharges (Chatterjee et al., 2007; Dominguez et al., 2010; Balu et al., 2020). Chatterjee et al. (2007) reported extensive use of wood and coal for home cooking, making molasses, and husking rice in the Sundarbans area.

**Table 5:** Loading factors, total and cumulative variance of pH, CaCO_3_, sand, silt, clay, and PAHs for LSHR, and SMW. In bold are the significant loads, p < 0.05

	LSHR			SMW		
	PC1	PC2	PC3	PC1	PC2	PC3
PH	−0.15	−0.33	0.22	−0.03	0.07	0.43
% OC	0.18	0.18	−0.30	0.28	0.21	−0.02
% CACO_3_	0.32	0.00	−0.30	−0.10	−0.42	−0.29
% Sand	0.08	−0.04	0.65	0.34	0.61	0.45
% Silt	0.02	0.23	−0.60	−0.24	0.01	0.32
% Clay	−0.05	−0.25	0.47	−0.22	−0.62	−0.62
NAP	0.19	−0.55	−0.53	**0.91**	0.12	−0.23
ACY	**0.88**	0.24	0.27	**0.94**	0.23	−0.16
FLR	0.40	**−0.82**	−0.17	−0.03	0.13	0.19
PHE	0.42	−0.58	−0.59	0.68	−0.59	0.37
ANT	0.51	−0.58	0.45	0.61	−0.45	−0.03
FLT	**0.73**	−0.62	0.01	**0.70**	−0.62	0.33
PYR	**0.82**	−0.50	0.13	**0.82**	−0.50	0.26
BaA	**0.80**	−0.15	0.42	**0.99**	0.07	−0.05
CHR	**0.93**	0.07	0.27	**0.99**	0.07	−0.06
BbF	**0.82**	0.51	−0.08	**0.96**	0.23	−0.12
BkF	**0.83**	0.51	−0.07	**0.97**	0.20	−0.14
BaP	**0.79**	0.49	−0.17	**0.96**	0.21	−0.15
DhA	0.03	0.06	0.11	0.24	0.41	0.21
IPY	**0.77**	0.47	−0.12	**0.96**	0.22	−0.14
BgP	**0.78**	0.50	−0.10	**0.96**	0.22	−0.15
TOT PAHs	**0.96**	−0.23	0.02	**0.98**	−0.17	0.08
TOT PD	**0.88**	0.42	−0.04	**0.97**	0.17	−0.13
LPAHs	0.61	−0.65	−0.38	**0.83**	−0.50	0.24
HPAHs	**0.98**	−0.15	0.08	**0.99**	−0.11	0.05
% total variance	41.93	18.19	10.69	57.05	11.98	6.49
% cumulative	41.93	60.13	70.81	57.05	69.03	75.52

### Potential toxicological assessment

Table 6 shows the values of TEQ, MEQ, CEQ and PAHs carc/ΣPAH. Mean TEQ was 285 ng/g and varied between 35.2 and 524 ng/g, with a maximum TEQ in the LSHR, S1, 524 ng/g, and S2, 390 ng/g. Lower TEQ was determined in the Sundarbans, with the value of 35.2 ng/g, S3, and 191 ng/g, S4. Our mean TEQ values appeared to be of the same order of magnitude as those reported by (Zuloaga et al., 2013) for the Sundarbans wetland in Bangladesh, 221 ng/g, and for the Sundarbans wetland in India, 358 ng/g. However, our TEQ range, 35.2–524 ng/g, appeared significantly lower than that of Bangladesh, 1.3–1,014 ng/g, and the Indian wetland, 1.3–2,450 ng/g (Zuloaga et al., 2013). Similarly to TEQ, MEQ appeared higher in LSHR, 56.8–312 ng/g, than in SMW, 37.5–233 ng/g. Mean TEQ and MEQ values resulted in several 100-fold in those relative to moderately polluted sites (Adeniji et al., 2019a, 2019b). The overall mean share of carcinogenic compounds was 21%, with a significantly higher mean share of 25% in LSHR than in Sundarbans, 16%.

**Table 6:** TEQ, MEQ, CEQ, and share of carcinogenic PAHs in PAHs (ng/g)

Site	TEQ	MEQ	CEQ	PAHs carc/ΣPAH
S1	524	56.8	0.29	0.12
S2	390	312	1.71	0.38
S3	35.2	37.5	0.13	0.18
S4	191	233	1.04	0.15
Mean	285	159	0.79	0.21
Min	35.2	37.5	0.13	0.12
Max	524	312	1.71	0.38

### Risk assessment of PAHs

The ecological risk of PAHs was determined by the calculation of the toxic equivalency factors based on the negligible concentrations, NCs, and maximum permissible concentrations, MPCs (Cao et al., 2010; Wu et al., 2011).

Table 7 lists the RQ (NCs) and RQ (MPCs) values of PAHs (ng/g) in the studied sediments, which were compared with the class of risk for individual hydrocarbons, free, low, moderate, high, and mix of ∑PAHs (Cao et al., 2010). In general, the risk of individual isomers was moderate, with values ≥ 1 except for a risk-free scenario detected for CHR, BbF, BkF, BaP, DhA, and IPYBgP in S3. The risk of the PAHs mix was high at all locations, being ∑PAHs relative to MPCs significantly higher than the threshold of 800, and this was particularly evident in S1, 3,091, and S2, 8034.

**Table 7:** Risk quotients of PAHs, to negligible concentrations, NCs, and maximum permissible concentrations, MPCs

PAH	S1	S2	S3	S4	NCs
NAP	1.86	0.377		2.46	1.4
ACY		38.7	0.968	30.0	1.2
ACE					1.2
FLR	2.04	2.60		0.375	1.2
PHE	50.5	28.7	30.1	20.6	5.1
ANT	49.9	90.7	61.5	52.5	1.2
FLT	16.0	28.4	14.0	9.84	26
PYR	179	483	154	142	1.2
BaA	4.68	103	6.13	22.2	3.6
CHR	0.218	2.82	0.291	0.877	107
BbF	3.29	69.2	5.41	42.3	3.6
BkF	0.293	6.70	0.423	3.59	24
BaP	0.624	4.21	0.689	3.90	27
DhA	3.71	1.43	0.057	0.265	27
IPY	0.188	2.89	0.308	2.46	59
BgP	0.136	2.35	0.318	1.78	75
ΣPAHs	312	866	274	335	
	S1	S2	S3	S4	MPCs
NAP	18.6	3.77		24.6	140
ACY		387	9.68	300	120
ACE					120
FLR	20.4	26.0		3.75	120
PHE	505	287	301	206	510
ANT	499	907	4615	525	120
FLT	160	284	140	98.4	2600
PYR	1785	4834	1540	1418	120
BaA	46.8	1030	61.3	222	360
CHR	2.18	28.2	2.91	8.77	10,700
BbF	3.29	69.2	5.41	42.3	3600
BkF	2.93	67.0	4.23	35.9	2400
BaP	6.24	42.1	6.89	39.0	2700
DhA	37.1	14.3	0.57	2.65	2700
IPY	1.88	28.9	3.08	24.6	5900
BgP	1.36	23.5	3.18	17.8	7500
ΣPAHs	3091	8034	2693	2969	

## Conclusions

The comparison of the PAHs presence in the surface sediments of the highly anthropized area of the Hooghly River and the natural area of the Sundarbans wetland revealed different loads, composition, sources, and ecotoxicological patterns of hydrocarbons. According to the data collected, the wetland looked less contaminated inside the wetland and moderately to highly polluted along the river. Additionally, it indicated that both sites were more abundant in high molecular PAHs, which pose a far greater risk to the ecosystem due to their higher mutagenic and carcinogenic potential than low molecular PAHs. The primary sources of PAHs in sediments are combustion from local car emissions, biomass burning, and home and industrial coal combustion, according to the use of diagnostic source ratios. But, in certain locations, a pyrogenic and petrogenic pattern was also noted, most likely because of the combination of urban and industrial discharges. Data provide background knowledge that will help create plans for the environmental protection of this vast wetland. When a comparison was made with SQGs, it became evident that the PAHs content in the Hooghly River site S2 was very high and this might likely affect aquatic biota, particularly benthic species, and necessitates the urgent implementation of preventive measures. This was also confirmed by the high ecological risk detected for PAHs mix, significantly higher than literature thresholds. Subsequent studies will demonstrate how monsoon cycling affects the temporal evolution of pollutant loads and the geographical distribution of PAHs in various HRE and SMW segments.

## Data Availability

The datasets generated during and/or analysed during the current study are available from the corresponding author on reasonable request.

## Abbreviations

PAHs: Polycyclic aromatic hydrocarbons
HR: Hooghly River
SMW: Sundarbans Mangrove Wetland
HRE: Hooghly River Estuary
LSHR: Last stretch of the Hooghly River
LOD: Low detection limit
LOQ: Limit of quantification
TOC: Total organic carbon
Sk_I_: Skewness
K_G_: Kurtosis
Mz: Mean grain size
PEL: Probable Effects Levels
ERM: Effects Range-Median
m-ERM-Q: Mean ERM quotient
MSQ: Marine Standard Quality
SQGs: Standard Quality Guidelines
NCs: Negligible concentrations
MPCs: Maximum permissible concentrations
RQ: Risk quotient
